# Monoclonal gammopathy of undetermined significance and COVID-19: a population-based cohort study

**DOI:** 10.1038/s41408-021-00580-7

**Published:** 2021-12-01

**Authors:** Saemundur Rognvaldsson, Elias Eythorsson, Sigrun Thorsteinsdottir, Brynjar Vidarsson, Pall Torfi Onundarson, Bjarni A. Agnarsson, Margret Sigurdardottir, Ingunn Thorsteinsdóttir, Isleifur Olafsson, Hrafnhildur L. Runolfsdottir, Dadi Helgason, Arna R. Emilsdottir, Arnar S. Agustsson, Aron H. Bjornsson, Gudrun Kristjansdottir, Asdis Rosa Thordardottir, Olafur Skuli Indridason, Asbjorn Jonsson, Gauti Kjartan Gislason, Andri Olafsson, Hlif Steingrimsdottir, Petros Kampanis, Malin Hultcrantz, Brian G. M. Durie, Stephen Harding, Ola Landgren, Runolfur Palsson, Thorvarður Jon Love, Sigurdur Yngvi Kristinsson

**Affiliations:** 1 Faculty of Medicine, University of Iceland, Reykjavik, Iceland; 2 Landspítali–The National University Hospital of Iceland, Reykjavik, Iceland; 3 Rigshospitalet, Copenhagen, Denmark; 4 Akureyri Hospital, Akureyri, Iceland; 5 The Binding Site ltd, Birmingham, UK; 6 Memorial Sloan-Kettering Cancer Center, New York City, NY USA; 7 Cedar-Sinai Samual Oschin Cancer Center, Los Angeles, CA USA; 8 Sylvester Cancer Center, University of Miami, Miami, FL USA

**Keywords:** Epidemiology, Risk factors, Myeloma, Infectious diseases

## Abstract

Multiple myeloma (MM) patients have increased risk of severe coronavirus disease 2019 (COVID-19) when infected by severe acute respiratory syndrome coronavirus 2 (SARS-CoV-2). Monoclonal gammopathy of undetermined significance (MGUS), the precursor of MM has been associated with immune dysfunction which may lead to severe COVID-19. No systematic data have been published on COVID-19 in individuals with MGUS. We conducted a large population-based cohort study evaluating the risk of SARS-CoV-2 infection and severe COVID-19 among individuals with MGUS. We included 75,422 Icelanders born before 1976, who had been screened for MGUS in the Iceland Screens Treats or Prevents Multiple Myeloma study (iStopMM). Data on SARS-CoV-2 testing and COVID-19 severity were acquired from the Icelandic COVID-19 Study Group. Using a test-negative study design, we included 32,047 iStopMM participants who had been tested for SARS-CoV-2, of whom 1754 had MGUS. Among these participants, 1100 participants, tested positive, 65 of whom had MGUS. Severe COVID-19 developed in 230 participants, including 16 with MGUS. MGUS was not associated with SARS-CoV-2 infection (Odds ratio (OR): 1.05; 95% confidence interval (CI): 0.81–1.36; *p* = 0.72) or severe COVID-19 (OR: 0.99; 95%CI: 0.52–1.91; *p* = 0.99). These findings indicate that MGUS does not affect the susceptibility to SARS-CoV-2 or the severity of COVID-19.

## Introduction

Severe acute respiratory syndrome coronavirus 2 (SARS-CoV-2) was first detected in Wuhan, China in 2019 [1], and has since developed into a global pandemic. The clinical presentation of the associated coronavirus disease 19 (COVID-19) varies from mild disease to multi-organ failure and death [2]. Risk factors for severe COVID-19 have been identified, including age, male sex, and several comorbidities including cancer [3].

Patients with multiple myeloma (MM), a malignancy of bone marrow plasma cells, are at a particularly high risk of developing severe illness when infected by SARS-CoV-2 [4–7]. Potential pathobiological mechanisms have been suggested, including immunosuppressive therapy, inherent suppression and dysregulation of humoral and cellular immunity, and MM-associated kidney disease. Currently, high disease burden, and severe hypogammaglobulinemia have been associated with increased risk for severe COVID-19 in MM patients, whilst treatement-related factors have not [4–7].

The precursor condition of MM, monoclonal gammopathy of undetermined significance (MGUS) [8, 9] is characterized by the presence of monoclonal immunoglobulins (M proteins) or free light chains (FLC) in the serum without MM-defining clinical or biological markers [10]. MGUS is common, affecting 4.2% of the general population over 50 years of age [11, 12]. MGUS has been associated with a similar but milder inherent immune dysfunction as MM, including significant defects in both humoral and cellular immunity [13] and relatively high rates of hypogammaglobulinemia (25%) [11, 14]. Furthermore, MGUS has been associated with a two-fold risk of bacteremia and almost three-fold risk of viral infections [15]. MGUS has also been associated with thrombosis [16] and kidney disease [17], both of which are features of and risk factors for severe COVID-19 [3]. Therefore, it has been speculated that individuals with MGUS might have an increased risk of SARS-CoV-2 infection and severe COVID-19 [18]. In a recently reported small (*n* = 7) case series of individuals with MGUS who were infected with SARS-CoV-2, five required hospitalization and one died [19]. However, no systematic data on MGUS and COVID-19 have been published to date.

MGUS is usually asymptomatic and is most often diagnosed incidentally during evaluation of unrelated medical problems. This leads to a biased selection of individuals with other comorbidities that may generate false associations between MGUS and various diseases. In fact, a previous study of a screened MGUS cohort in the US could not confirm many of the disease associations found in clinical cohorts [20], highlighting the need to use screened MGUS cohorts to assess the association of MGUS with other diseases, including COVID-19.

Here, we report the first study of COVID-19 in individuals with MGUS using data from the ongoing Iceland Screens Treats or Prevents Multiple Myeloma study (iStopMM), which has already screened 75,422 Icelanders for MGUS. The objective of the study was to evaluate the risk of SARS-CoV-2 infection and severe COVID-19 in individuals with MGUS. Based on the previous literature we hypothesized that MGUS might increase the risk of SARS-CoV-2 infection and severe COVID-19.

## Methods

### Ethical approval

The iStopMM study, data collection for Icelandic patients with SARS-CoV-2 infection, and crosslinking of healthcare data has been approved by the Icelandic National Bioethics Committee (VSN 16-022, date: 2016-04-26; VSN 20-078, date: 2021-05-26) with additional approval from the Icelandic Data Protection Agency. The iStopMM study has been registered on ClinicalTrials.gov (ClinicaTrials.gov identifier: NCT03327597).

### COVID-19 in Iceland

The first case of SARS-CoV-2 infection in Iceland was diagnosed on February 28, 2020. The Icelandic authorities implemented an aggressive testing strategy early in the pandemic that included targeted testing based on clinical suspicion, open invitation population screening, and random screening for SARS-CoV-2 among asymptomatic persons [21]. As the pandemic continued, random screening was discontinued, while self-ordered testing with same-day results became available to all and double screening of individuals in quarantine and persons arriving at the border was initiated. All SARS-CoV-2 testing was done by real-time quantitative polymerase chain reaction (qPCR) of simultaneously acquired oropharyngeal and nasopharyngeal swabs. Through this approach, Iceland has consistently ranked among the nations with the highest level of testing in the world. In total, 6,126 individuals were found to be SARS-CoV-2-positive by qPCR in Iceland between February 28 and December 31, 2020 [22].

All SARS-CoV-2-positive individuals were centrally registered and immediately contacted by the COVID-19 Outpatient Clinic at Landspitali–The National University Hospital at the time of diagnosis. If the diagnostic sample was obtained during asymptomatic screening, a repeat qPCR test of a nasopharyngeal sample and a blood test for SARS-CoV-2-antibodies were performed within 24 h. All persons who were considered to have an active SARS-CoV-2 infection were isolated and enrolled into telehealth monitoring at the COVID-19 Outpatient Clinic. Monitoring consisted of serial telephone interviews conducted by either a nurse or physician using a standardized data entry form. Patients reporting concerning symptoms were evaluated at the clinic and admitted to the hospital if needed. Patients were monitored for at least 14 days after their first positive qPCR and until they had been asymptomatic for at least seven days. This comprehensive systematic approach of combined community and clinical care has previously been described in detail [23].

### Study cohort

The study cohort was comprised of participants in the iStopMM study who had been screened for MGUS before December 31, 2020 and were alive and had not been diagnosed with MM and related disorders, including smoldering MM requiring treatment, before February 28, 2020. iStopMM is a population-based screening study for MGUS and randomized trial of follow-up strategies in Iceland. All Icelanders born in 1975 (*n* = 148,704) and earlier were invited to participate and 80,759 (54%) accepted and provided informed consent between September 2016 and February 2018. Serum samples were collected from 75,422 study participants (93%) between September 9, 2016 and December 31 2020 and screened for MGUS by capillary zone electrophoresis (CZE), immunofixation electrophoresis (IFE), and serum FLC assay. The iStopMM study design has previously been described in detail [24].

### Study design and statistical analysis

The primary exposure was MGUS as determined by M protein detectable on CZE and confirmed by IFE or an abnormal FLC ratio (kappa/lambda ratio <0.26 and lambda >26.3 g/L or a kappa/lambda ratio >1.65 and kappa >19.4 g/L). Those with MGUS were further subdivided into heavy chain-MGUS (HC-MGUS) and light-chain MGUS (LC-MGUS) subgroups. All analyses were carried out separately for MGUS, HC-MGUS, and LC-MGUS. When included as a covariate, age was modeled with a four-knot restricted cubic spline.

In the first analysis, we evaluated whether there was an association between MGUS and testing positive for SARS-CoV-2 using a test-negative study design. Participants from the study cohort who had been tested at least once for SARS-CoV-2 between February 28 and December 31, 2020, were included. Those who had at least one positive qPCR test for SARS-CoV-2 were considered to be infected. The association of MGUS and SARS-CoV-2 infection was evaluated using logistic regression adjusting for sex and age.

In the second analysis, we evaluated the association between MGUS and severe COVID-19. Participants from the previous analysis who tested positive for SARS-CoV-2 were included. Those who were hospitalized for other medical problems or were living in a nursing home at the time of testing were excluded. Participants were followed until discharge from telehealth monitoring or until they had developed severe COVID-19. Severe COVID-19 was defined as the composite outcome of requiring an emergency outpatient visit, requiring hospital admission, or death (emergency outpatient visit or worse). Additionally, we conducted an analysis where severe COVID-19 was defined as hospital admission or death (hospital admission or worse). The association of MGUS with severe COVID-19 was assessed using logistic regression, adjusting for sex and age.

Two sensitivity analyses were performed. In the first sensitivity analysis, we evaluated the association of MGUS and SARS-CoV-2 testing in the whole study cohort using logistic regression, adjusting for sex and age. In the second sensitivity analysis, we repeated the first analysis and included the entire study cohort regardless of whether the participants had been tested for SARS-CoV-2 or not.

All analyses were carried out in R, version 3.6.3, using the *rms* package [25]. The code used for this study and its output have been published online at https://osf.io/kfdg9/.

## Results

**Fig. 1:**
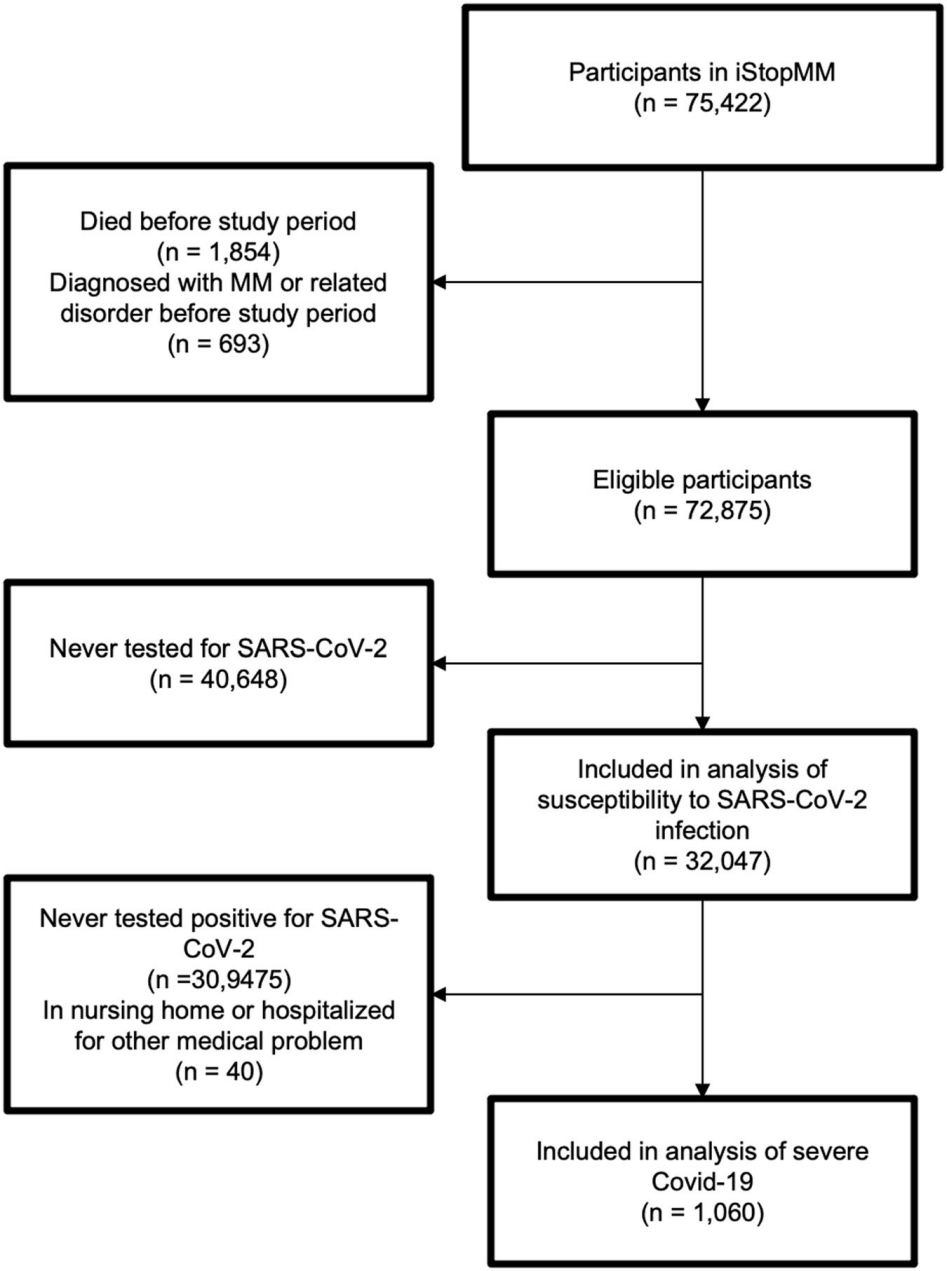
Participant selection. Flowchart demonstrating the inclusion and exclusion of participants.

**Fig. 2:**
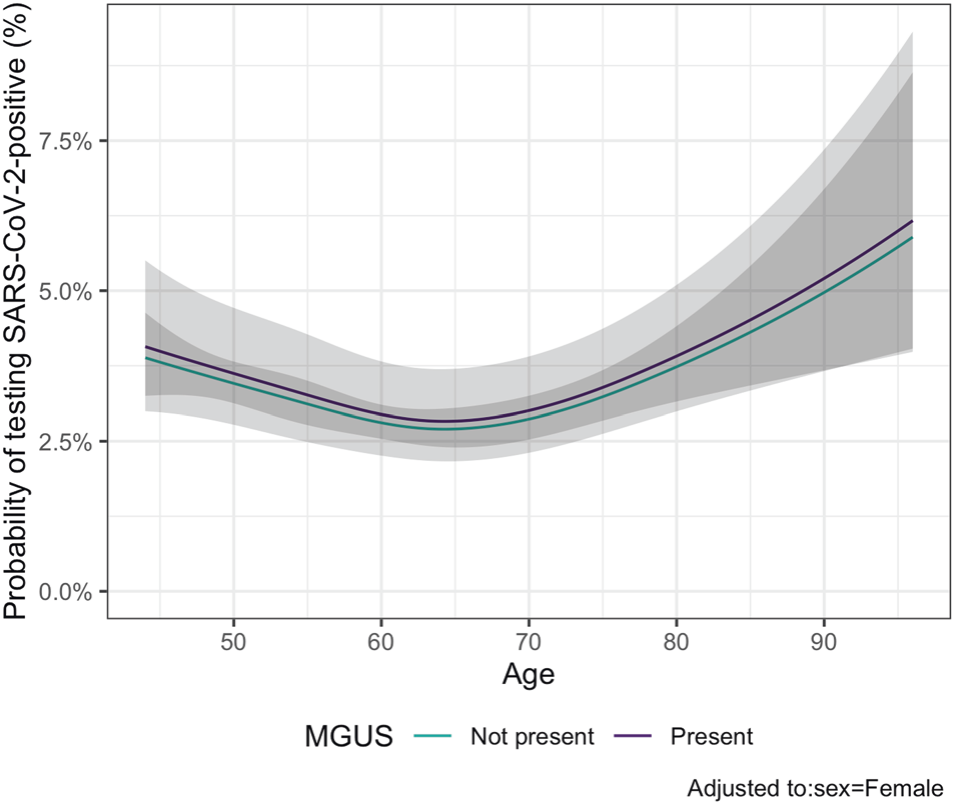
Probability of testing SARS-CoV-2 positive. Probability of individuals with or without MGUS of testing positive for SARS-COV-2 according to age, adjusted to female sex.

Of the 75,422 participants who had been screened for MGUS, 1,854 had died and 693 had been diagnosed with MM and related disorders before the study period. A total of 32,047 participants, of whom 1,754 (5.5%) had MGUS, had been tested for SARS-CoV-2 (Fig. 1). Those who had MGUS were older (mean age 66.3 vs 59.1 years, *p* < 0.001) and more likely to be male (50% vs 41%, *p* < 0.001) than those who did not have MGUS. Of those tested, 1,100 (3.4%) were positive for SARS-CoV-2, including 65 who had MGUS. After adjusting for sex and age, MGUS was not found to be associated with SARS-CoV-2 infection (odds ratio (OR): 1.05; 95% confidence interval (CI): 0.81–1.36; *p* = 0.72). The findings were similar for HC- and LC-MGUS (Table 1 and Fig. 2). Sensitivity analysis that included the whole study chort showed essentially the same results (Supplemental Table). There was no significant association between MGUS and the rate of SARS-CoV-2 testing (Supplemental Table).

**Table 1:** Baseline characteristics of study participants tested for SARS-CoV-2 and the association between MGUS and SARS-CoV-2 infection.

	No MGUS	MGUS	HC-MGUS	LC-MGUS
*n*	30,293	1754	1140	614
Mean age (SD)	59 (10)	66 (11)	65.9 (11)	66.9 (11)
Men	12,283 (41%)	880 (50%)	571 (50%)	309 (50%)
Person-years	24,882	1431	–	–
SARS-CoV-2-positive	1035 (3.4%)	65 (3.7%)	41 (3.6%)	24 (3.9%)
OR of SARS-CoV-2 positivity (95% CI)^a^	Ref	1.05 (0.80-1.36)	1.02 (0.74-1.40)	1.11 (0.73-1.69)

*MGUS* monoclonal gammopathy of undetermined significance, *HC-MGUS* heavy chain MGUS, *LC-MGUS* light chain MGUS, *SD* standard deviation, COVID-19 coronavirus disease 2019, *OR* odds ratio, *CI* confidence interval, *Ref* reference.

^a^ Adjusted for age and sex.

**Fig. 3:**
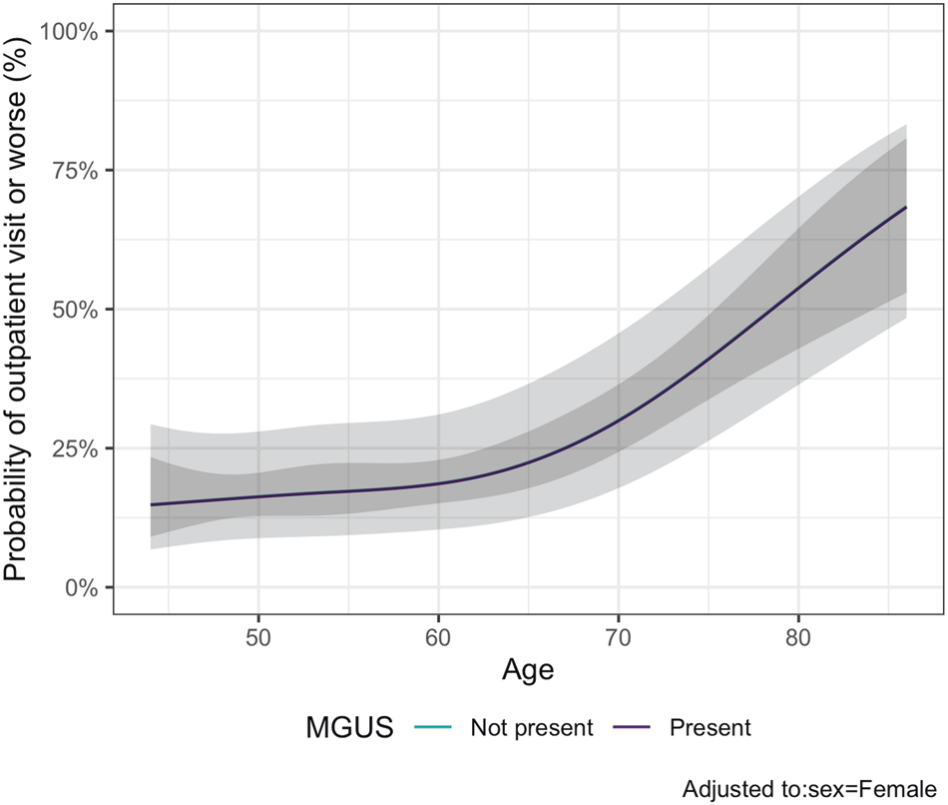
Probability of severe COVID-19. Probability of individuals with or without MGUS having severe COVID-19 according to age, adjusted to female sex. Severe COVID-19 is defined as the composite outcome of emergency outpatient visits, hospital admission, or death.

Among 1100 persons who tested positive for SARS-CoV-2, 40 were hospitalized for other medical problems or were residing in a nursing home at the time of SARS-CoV-2 testing (Fig. 1). Of the remaining 1060 cases, 56 had MGUS. During follow-up, 16 (29%) individuals with MGUS and 214 (21%) without the disorder developed severe COVID-19. We did not find MGUS to be associated with severe COVID-19 when defined as emergency outpatient visit or worse (OR: 0.99; 95%CI: 0.52–1.91; *p* = 0.99) or as hospital admission or worse, (OR: 1.13; 95%CI: 0.52–2.46; *p* = 0.76). The findings were similar for HC- and LC-MGUS (Table 2 and Fig. 3).

**Table 2:** Baseline characteristics of SARS-CoV-2-positive participants and the association between MGUS and severity of COVID-19.

	No MGUS	MGUS	HC-MGUS	LC-MGUS
*n*	1004	56	35	21
Mean age (SD)	59 (10)	65 (11)	65 (12)	65 (10)
Men	448 (45%)	30 (54%)	22 (63%)	8 (38%)
Person-days	16,589	962	–	–
Emergency outpatient visit	176 (18%)	12 (20%)	7 (20%)	5 (24%)
Hospital admission	105 (11%)	11 (20%)	8 (23%)	3 (14%)
Intensive care unit admission	20 (2%)	3 (5%)	2 (6%)	1 (5%)
Death	5 (1%)	0 (0%)	0 (0%)	0 (0%)
Emergency outpatient visit or worse	214 (21%)	16 (29%)	10 (29%)	6 (29%)
OR (95% CI)^a^	Ref	0.99 (0.52–1.91)	0.90 (0.39–2.08)	1.10 (0.39–3.10)
Hospital admission or worse	106 (11%)	11 (20%)	8 (23%)	3 (14%)
OR (95% CI)^a^	Ref	1.13 (0.52–2.46)	1.25 (0.49–3.19)	0.83 (0.21–3.29)

*MGUS* monoclonal gammopathy of undetermined significance, *HC-MGUS* heavy chain MGUS, *LC-MGUS* light chain MGUS, *SD* standard deviation, *COVID-19* Coronavirus disease 2019, *OR* odds ratio, *CI* confidence interval, *Ref* reference.

^a^ Adjusted for age and sex.

## Discussion

This large nationwide cohort study is the first population-based study evaluating COVID-19 susceptibility and severity among persons with MGUS. Although previous authors have speculated that MGUS may impact the risk of SARS-CoV-2 infection and especially the risk of severe COVID-19 [18], we did not find MGUS to be associated with contracting SARS-CoV-2 or developing severe COVID-19 once infected.

MGUS was not associated with an increased risk of SARS-CoV-2 infection. This contradicts previous studies that have found individuals with MGUS to have an increased risk of infections, including viral infections [15]. This might indicate that this increased risk of viral infections does not apply to COVID-19. However, these studies were based on individuals with incidentally diagnosed MGUS, which may have biased the sample to include individuals with more comorbid conditions. By contrast, our study is based on a cohort of persons who were screened for MGUS and underwent a high rate of SARS-CoV-2 testing (42% being tested at least once) and no difference in the frequency of testing between those with and without MGUS. This minimizes the possibility of detection bias due to MGUS in this study.

Individuals with MGUS did not have an increased risk of severe COVID-19 compared to those without MGUS. This is in contrast to MM, which has been associated with severe COVID-19 [4–7]. Severe COVID-19 is believed to be caused by immune dysregulation and hyperactivation [26]. Disease factors rather than treatment factors in MM have been associated with severe COVID-19 and it has been speculated that immune dysregulation inherent in MM increases the risk of severe COVID-19 [4–7]. It is therefore unexpected that we did not find an increased risk of severe COVID-19 in individuals with MGUS, who have been shown to have similar, although milder, dysregulation of humoral and cellular immune function [13]. These findings indicate that more severe immune dysregulation, as seen in MM, contributes to the risk of severe COVID-19 or that the effects of treatment factors have been underestimated in previous studies.

This study has several strengths. Firstly, the entire study population was screened for MGUS, thereby eliminating the selection bias present in most other MGUS cohorts where disorder is primarily diagnosed in those who have other medical problems and are therefore likely to have a greater burden of comorbid conditions. Furthermore, the non-MGUS group had been tested for MGUS removing potential false negatives from that study group. Secondly, the comprehensive and aggressive SARS-CoV-2 testing strategy employed in Iceland has yielded a high case capture rate compared to other nations. Thirdly, data on diagnosis and follow-up of COVID-19 cases, which has been collected by the same clinical team, are virtually complete, and centrally registered for the whole nation. Finally, the study included a large number of persons who represent a significant proportion of the nation’s population making the findings likely to be generalizable to similar populations.

This study also has limitations. Firstly, we included individuals based on blood testing alone which does not completely exclude more advanced diseases than MGUS. However, those who had MM or related disease, including smoldering MM in treatment, were excluded. Secondly, despite being the largest study to date with more than 75,422 participants, the number of events was relatively low, and therefore, the study might be underpowered to detect a modest increase in risk. Thirdly, due to the relative scarcity of participants with MGUS and SARS-CoV-2 infection and the low mortality from COVID-19 in Iceland, hard endpoints were too rare for this analysis requiring the use of composite outcomes. Finally, the Icelandic population is highly genetically homogenous and mostly white, limiting generalization in non-white populations.

In conclusion, in this large population-based cohort study including 75,422 individuals screened for MGUS, we did not find MGUS to be a risk factor for contracting SARS-CoV-2 or developing severe COVID-19. These findings are important since MGUS is the most common precursor condition of hematological malignancy, affecting millions of individuals worldwide [11, 12]. The findings provide guidance for how physicians should counsel their patients with MGUS about their risks during the COVID-19 pandemic.

## Supplementary information

Supplemental table

STROBE checklist

AJ checklist
