# Supplementary material for: Monoclonal gammopathy of undetermined significance and COVID-19: a population-based cohort study
Source: Blood Cancer J. 2021 Dec 1;11(12):191. doi: 10.1038/s41408-021-00580-7 (PMC8635472; doi:10.1038/s41408-021-00580-7)
Supplement: Supplementary file 1 — Supplemental table [file 41408_2021_580_MOESM1_ESM.docx]

**Supplemental Table:** Baseline characteristics of the whole study cohort and the association between MGUS and SARS-CoV-2 infection, independent of testing.

|  | **Not MGUS** | **MGUS** | **HC-MGUS** | | **LC-MGUS** |
| --- | --- | --- | --- | --- | --- |
| *n* | 67,829 | 5,046 | 3,228 | 1,818 | |
| *Mean age (SD)* | 62 (11) | 70 (11) | 70 (11) | 71 (11) | |
| *Men* | 30,068 (44%) | 2,702 (54%) | 1,709 (53%) | 993 (55%) | |
|  |  |  |  |  | |
| *Tested for SARS-CoV-2* | 30,293 | 1,754 | 1140 | 614 | |
| *OR (95% CI)** | Ref | 1.01 (0.95-1.08) | 1.02 (0.94-1.10) | 1.00 (0.90-1.10) | |
|  |  |  |  |  | |
| *SARS-CoV-2-positive* | 1035 (3.4%) | 65 (3.7%) | 32 (3.3%) | 18 (3.6%) | |
| *OR (95% CI)** | Ref | 1.06 (0.82-1.37) | 1.04 (0.75-1.43) | 1.11 (0.74-1.68) | |

*Adjusted for age and sex.

Abbreviations: MGUS: Monoclonal gammopathy of undetermined significance HC-MGUS: Heavy chain MGUS; LC-MGUS: Light chain MGUS; SD: Standard deviation; COVID-19: Coronavirus disease 2019; OR: Odds ratio; CI: Confidence interval.
